# Anticholinergic drugs and forebrain magnetic resonance imaging changes in cognitively normal people and those with mild cognitive impairment

**DOI:** 10.1111/ene.15251

**Published:** 2022-02-07

**Authors:** Dewen Meng, Ali‐Reza Mohammadi‐Nejad, Stamatios N. Sotiropoulos, Dorothee P. Auer, for the Alzheimer's Disease Neuroimaging Initiative

**Affiliations:** ^1^ 6123 Radiological Sciences, Mental Health & Clinical Neurosciences School of Medicine University of Nottingham Nottingham UK; ^2^ 6123 Sir Peter Mansfield Imaging Centre School of Medicine University of Nottingham Nottingham UK; ^3^ 6123 National Institute for Health Research Nottingham Biomedical Research Centre Queen's Medical Centre University of Nottingham Nottingham UK

**Keywords:** anticholinergic drugs, cognition, forebrain, functional connectivity, gray matter density, nucleus basalis of Meynert

## Abstract

**Background and purpose:**

Anticholinergic (AC) medication use is associated with cognitive decline and dementia, which may be related to an AC‐induced central hypocholinergic state, but the exact mechanisms remain to be understood. We aimed to further elucidate the putative link between AC drug prescription, cognition, and structural and functional impairment of the forebrain cholinergic nucleus basalis of Meynert (NBM).

**Methods:**

Cognitively normal (CN; *n* = 344) and mildly cognitively impaired (MCI; *n* = 224) Alzheimer’s Disease Neuroimaging Initiative Phase 3 participants with good quality 3‐T magnetic resonance imaging were included. Structural (regional gray matter [GM] density) and functional NBM integrity (functional connectivity [FC]) were compared between those on AC medication for > 1 year (AC^+^) and those without (AC^−^) in each condition. AC burden was classed as mild, moderate, or severe.

**Results:**

MCI AC^+^ participants (0.55 ± 0.03) showed lower NBM GM density compared to MCI AC^−^ participants (0.56 ± 0.03, *p* = 0.002), but there was no structural AC effect in CN. NBM FC was lower in CN AC^+^ versus CN AC^−^ (3.6 ± 0.5 vs. 3.9 ± 0.6, *p* = 0.001), and in MCI AC^+^ versus MCI AC^−^ (3.3 ± 0.2 vs. 3.7 ± 0.5, *p* < 0.001), with larger effect size in MCI. NBM FC partially mediated the association between AC medication burden and cognition.

**Conclusions:**

Our findings provide novel support for a detrimental effect of mild AC medication on the forebrain cholinergic system characterized as functional central hypocholinergic that partially mediated AC‐related cognitive impairment. Moreover, structural tissue damage suggests neurodegeneration, and larger effect sizes in MCI point to enhanced susceptibility for AC medication in those at risk of dementia.

## Graphical abstract

Central cholinergic systems effects of anticholinergic medication. The central cholinergic network as reconstructed from resting state fMRI (red‐yellow) is impaired in older participants on mild‐moderate anticholinergic medication compared to those without. The medication effect on the functional cholinergic disconnection is more pronounced in those with mild cognitive impairment (MCI). Only participants with MCI showed reduced gray matter density in the nucleus basalis of Meynert (blue). Our findings suggest enhanced vulnerability of the central cholinergic system to anticholinergic drugs in MCI.

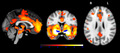

## INTRODUCTION

Cognition is known to depend on modulation of cortical activity by cholinergic innervation from the nucleus basalis of Meynert (NBM) [1]. Cholinergic neurons of the NBM are particularly vulnerable to Alzheimer disease (AD) and other neurodegenerative disorders [2, 3, 4]. The subsequent loss of cholinergic innervation plays an important role in the development of cognitive impairment [5, 6]. Mechanistic evidence for AD as a hypocholinergic syndrome comes from the therapeutic benefits of enhancing cholinergic neurotransmission by cholinesterase inhibitors (ChEIs) [7], with early beneficial results being reported from trials of deep brain stimulation of the NBM [8].

Further support of a hypocholinergic mechanism of cognitive decline comes from consistent observations of cognitive impairment after experimental exposure to anticholinergic (AC) drugs, with some evidence that AC drugs may accelerate amyloid deposition [9, 10]. Several epidemiological studies reported AC‐related increased risk of dementia [11, 12], which may account for 10% of dementia cases [12], equating to approximately 20,000 new cases of dementia per year in the UK alone. This has raised significant interest in possible deprescription trials [13] but there is limited mechanistic understanding of how AC drugs increase the risk of dementia in specific populations across the ageing–predementia continuum [14].

Neuroimaging studies allow investigation of the underpinnings of detrimental brain effects of centrally acting medication. Magnetic resonance imaging (MRI) studies have demonstrated NBM atrophy across the ageing–mild cognitive impairment (MCI)–AD continuum using a range of measurements [15, 16, 17, 18] with predictive power for the transition to AD [19] and cognitive decline in Parkinson disease (PD) [20]. An association of AC medication use with structural impairment of the NBM would provide suggestive evidence for cumulative tissue damage as a marker of accelerated neurodegeneration of the central cholinergic system. Functional markers of the forebrain cholinergic system, on the other hand, would be expected to more sensitively indicate inhibitory AC drug effects on the cholinergic circuit and to precede structural changes. NBM functional disconnection has been shown in preclinical states of AD [4, 21] in line with the expected hypocholinergic states due to NBM degeneration. Importantly, the combined use of functional and structural NBM markers can shed light onto the largely unexplored interplay between AC drugs and cognition in preclinical and prodromal AD.

The aim of this study was to investigate the link between AC medication use, cognitive decline, and structural and functional integrity of the NBM in a cohort of elderly cognitively normal (CN) and MCI participants. We hypothesized that participants with AC medication use compared to those without would (i) show NBM gray matter (GM) density loss as a marker of structural damage to NBM and (ii) display disrupted functional cholinergic networks as a marker of central hypocholinergic state. We also hypothesized (iii) that AC burden (ACB) is associated with cognitive impairment and that this association is partially mediated by NBM affection. We also report on secondary tests that include comparisons of AC medication effect size between MRI markers, and bias assessment by comparing clinical risk profiles and AD biomarkers between AC medication strata.

## METHODS

### Participants

Data used in the preparation of this article were obtained from the Alzheimer’s Disease Neuroimaging Initiative (ADNI) database (http://adni.loni.usc.edu). ADNI was launched in 2003 as a public–private partnership, led by principal investigator Michael W. Weiner, MD. The primary goal of ADNI has been to test whether serial MRI, positron emission tomography (PET), other biological markers, and clinical and neuropsychological assessment can be combined to measure the progression of MCI and early AD. This study was approved by the institutional review boards of all participating institutions. Written informed consent was obtained from all individuals.

A total of 417 CN and 332 MCI participants' (Appendix S1) T1‐weighted MRI and resting‐state functional MRI (rsfMRI) data were downloaded from ADNI3 (26 October 2019). Information on age, sex, medical history, and years of education was retrieved from the latest available dataset in ADNI documents. For cognition, we chose the Alzheimer's Disease Assessment Scale–Cognitive Subscale (ADAS‐Cog) [22] to assess cognitive function, including memory and executive function, that is linked with cholinergic function [23].

### AC medication exposure

Information on medications with potential AC effect was manually extracted from the ADNI concomitant medication file, and drugs were classified according to the most commonly used ACB scale [24]. An AC^+^ participant was defined as anyone taking any AC medication for at least 1 year [25] until enrolled in the ADNI study. AC^−^ status was defined as no documented exposure to any AC drug for any duration. The total ACB score (TACB) was the sum of ACB scores (low [ACB = 1], medium [ACB = 2], or high [ACB = 3]) of all medication prescribed to each participant. We excluded participants who took antiparkinsonian medication with AC effects to avoid inclusion of prodromal PD pathology with known NBM degeneration [20]. Participants who took ChEIs were also excluded from this study.

### MRI data

Three‐ Tesla sagittal T1‐weighted images and axial rsfMRI data were used. We selected participants who have both T1‐weighted images and rsfMRI data. Three‐dimensional magnetization‐prepared rapid acquisition gradient echo images (echo time [TE] = minimum full echo, repetition time [TR] = 2300 ms, inversion time = 900 ms, field of view [FOV] = 208 × 240 × 256 mm, resolution = 1 × 1 × 1 mm^3^) and rsfMRI data (eyes open, ADNI3 Basic Protocol: TE = 30 ms, TR = 3000 ms, flip angle = 90°, FOV = 220 × 220 × 163 mm^3^, resolution = 3.4 × 3.4 × 3.4 mm^3^, 10 min) were downloaded. To avoid protocol bias effects, resting‐state ADNI3 Advanced Protocol data were excluded.

**FIGURE 1:**
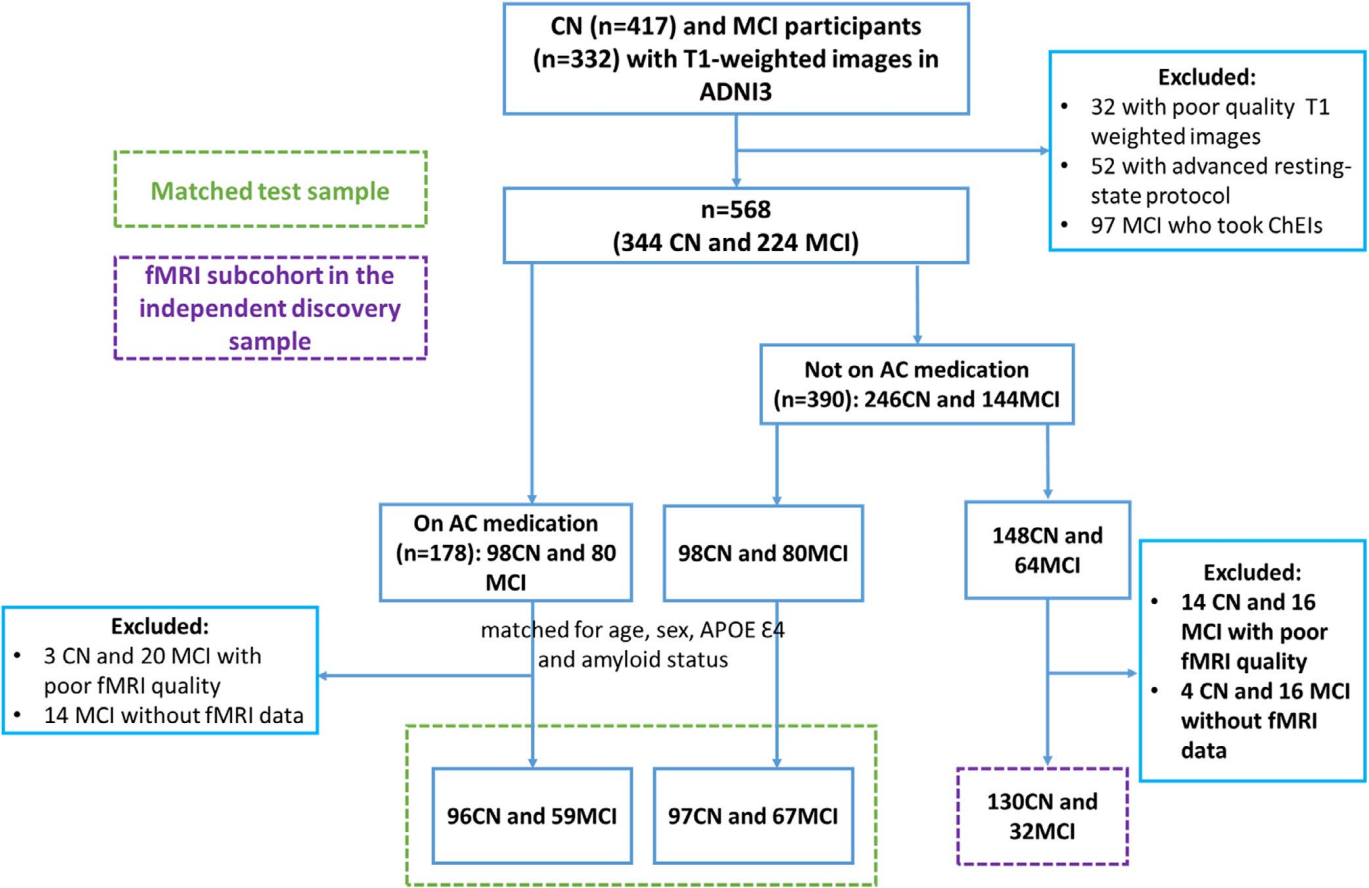
Flowchart shows participant selection. AC, anticholinergic medication; ADNI3, Alzheimer’s Disease Neuroimaging Initiative Phase 3; ChEI, cholinesterase inhibitor; CN, cognitively normal; fMRI, task‐free functional MRI; MCI, mild cognitive impairment [Colour figure can be viewed at wileyonlinelibrary.com]

A comprehensive structural and functional MRI pipeline (https://github.com/SPMIC‐UoN/BRC_Pipeline) that uses FSL tools (www.fmrib.ox.ac.uk/fsl) was used to quality control and preprocess the MRI data. This resulted in a final dataset of 568 participants for structural MRI, including 363 for rsfMRI. Figure 1 shows the flowchart of participant selection.

To reduce risk of comorbidity bias in the AC medication use cases, we undertook manual sample matching at the subgroup level to identify the equivalent number of AC^+^ to AC^−^ matched for age, sex, *APOE* Ɛ4, and amyloid status in the CN and MCI subcohorts (Table 1). Our matched test sample included 193 CN and 126 MCI participants who had both T1‐weighted images and rsfMRI data. The remainder subsample of rsfMRI participants not included in the matched AC^+^ and AC^−^ test sample (130 CN and 32 MCI participants, no AC^+^) provided an independent discovery sample to reconstruct the NBM functional connectivity (FC) network template.

**TABLE 1:** Demographic, clinical, and cognitive information in the matched test sample (age, sex, *APOE* Ɛ4, and amyloid status matched)

Characteristic	CN, *n* = 193				MCI, *n* = 126				*p* ^c^
	Total, *n* = 193	AC^−^ participants, *n* = 97	AC^+^ participants, *n* = 96	*p* ^a^	Total, *n* = 126	AC^−^ participants, *n* = 67	AC^+^ participants, *n* = 59	*p* ^b^	
Age, mean (SD), years	74.4 (8.2)	73.7 (8.5)	75.1 (7.9)	0.258	74.2 (8.1)	74.3 (8.2)	74.2 (8.2)	0.938	0.866
Female, *n* (%)	119 (61.7)	59 (60.8)	60 (62.5)	0.883	65 (51.6)	36 (53.7)	29 (49.2)	0.721	0.083
Education, mean (SD), years	16.7 (2.4)	17.0 (2.4)	16.4 (2.4)	0.092	16.5 (2.5)	16.8 (2.5)	16.2 (2.4)	0.240	0.595
**ADAS‐Cog, mean (SD)**	13.4 (4.9)	12.3 (4.1)	14.5 (5.4)	0.004*	16.2 (5.8)	15.8 (5.2)	16.6 (6.3)	0.472	<0.001*
*APOE* Ɛ4 carriers, *n* (%)^d^	35 (27.3)	20 (29.4)	15 (25.0)	0.692	23 (28.0)	12 (29.3)	11 (26.8)	0.500	0.517
Amyloid positive, *n* (%)^e^	49 (41.2)	27 (43.5)	22 (38.6)	0.709	28 (43.8)	15 (45.5)	13 (41.9)	0.806	0.756
**Vascular risk, CMC score, mean (SD)**	1.1 (1.0)	0.8 (0.8)	1.4 (1.1)	<0.001*	1.3 (0.9)	1.2 (0.9)	1.4 (0.9)	0.279	0.102
Total ACB score, median (range)	–	–	1 (1–7)	–	–	–	1 (1–7)	–	–
**Psychiatric**	54 (28.0)	21 (21.6)	33 (34.4)	0.055	50 (39.7)	22 (32.8)	28 (47.5)	0.104	0.038*
**Neurologic, other than cognitive disorder**	74 (38.3)	27 (27.8)	47 (49.0)	0.003*	69 (54.8)	32 (47.8)	37 (62.7)	0.108	0.006*
Head, eyes, ears, nose, throat	130 (67.4)	60 (61.9)	70 (72.9)	0.125	84 (66.7)	44 (65.7)	40 (67.8)	0.851	0.904
**Cardiovascular**	133 (68.9)	55 (56.7)	78 (81.3)	<0.001*	94 (74.6)	46 (68.7)	48 (81.4)	0.151	0.312
**Respiratory**	47 (24.4)	17 (17.5)	30 (31.3)	0.030*	42 (33.3)	16 (23.9)	26 (44.1)	0.023*	0.097
Hepatic	6 (3.1)	3 (3.1)	3 (3.1)	0.654	5 (4.0)	2 (3.0)	3 (5.1)	0.664	0.758
Dermatologic connective tissue	73 (37.8)	37 (38.1)	36 (37.5)	0.522	43 (34.1)	24 (35.8)	19 (32.2)	0.710	0.552
Musculoskeletal	151 (78.2)	73 (75.3)	78 (81.3)	0.384	94 (74.6)	49 (73.1)	45 (76.3)	0.838	0.498
Endocrine–metabolic	105 (54.4)	51 (52.6)	54 (56.3)	0.665	76 (60.3)	39 (58.2)	37 (62.7)	0.716	0.355
**Gastrointestinal**	109 (56.5)	47 (48.5)	62 (64.6)	0.029*	69 (54.8)	32 (47.8)	37 (62.7)	0.108	0.818
Hematopoietic–lymphatic	22 (11.4)	13 (13.4)	9 (9.4)	0.498	17 (13.5)	11 (16.4)	6 (10.2)	0.434	0.603
Renal–genitourinary	101 (52.3)	50 (51.5)	51 (53.1)	0.886	56 (44.4)	30 (44.8)	26 (44.1)	0.540	0.172
Allergies or drug sensitivities	75 (38.9)	32 (33.0)	43 (44.8)	0.105	48 (38.1)	23 (34.3)	25 (42.4)	0.365	0.907
Smoking, alcohol use, and/or drug use	17 (8.8)	5 (5.2)	12 (12.5)	0.081	20 (15.9)	7 (10.4)	13 (22.0)	0.091	0.073
**Malignancy**	44 (22.8)	18 (18.6)	26 (27.1)	0.173	14 (11.1)	8 (11.9)	6 (10.2)	0.785	0.008*

Abbreviations: AC, anticholinergic; ACB, AC burden; ADAS‐Cog, Alzheimer's Disease Assessment Scale–Cognitive Subscale; CMC, chronic metabolic conditions; CN, cognitively normal; MCI, mild cognitive impairment. Dominant characteristics are shown in bold.

**^a^P value of:** group comparison using *t*‐test between CN and MCI participants.

**^b^P value of:** group comparison using *t*‐test between AC^−^ CN and AC^+^ CN participants.

**^c^P value of:** group comparison using *t*‐test between AC^−^ MCI and AC^+^ MCI participants.

d In total, 210 participants had *APOE* Ɛ4 carrier information.

e In total, 183 participants had amyloid information.

* Significant at *p* < 0.05.

### NBM MRI metrics

The mean NBM GM density of each participant was estimated using an existing probabilistic anatomical map of Ch4 [26] available in SPM 12 Anatomy Toolbox [27] (Appendix S2) and established approach using FSL tools (Appendix S3).

To address the lack of an NBM network template and to overcome limitations of manual seed extraction [28] of the complex anatomical shape of NBM, we reconstructed an NBM FC network template using the NBM template as seed in the independent discovery sample. Details of NBM seed‐based FC analysis are provided in Appendix S3. As a marker of the functional integrity of the NBM network, we chose individual FC metrics derived as mean *Z* scores from individual NBM seed to NBM network maps using a priori defined seed and network templates.

### Bias assessment tests

We computed a score for chronic metabolic conditions (CMC) to reflect the systemic vascular health and dichotomized participants using the median score of 2 [29]. Participants were designated as *APOE* Ɛ4 carriers if they had one or two copies of allele 4, and as noncarriers if they had no allele 4 in their genotype. Participants with positive amyloid pathology were classified according to the semiquantitative amyloid β1–42 peptide PET results retrieved from the latest available dataset in ADNI documents (https://adni.loni.usc.edu).

We also extracted hippocampus volumes and precuneus GM volumes as established MRI markers of AD pathology from the latest ADNI documents, which were analysed by using the FreeSurfer image analysis suite (http://surfer.nmr.mgh.harvard.edu/). To control for nonspecific, such as vascular, effects of AC medication on FC metrics, we also compared FC changes in the visual cortex between AC strata using the primary visual cortex as a seed region for FC analysis.

### Statistical analysis

The statistical analyses were conducted using SPSS (v21). One‐way analysis of variance and *χ* ^2^ test were used to compare demographics, cognitive performance, AD risk markers, and CMC risk between AC^+^ and AC^−^ in CN and MCI, and between CN and MCI; the significance level was set at *p* < 0.05. For exploratory bias assessment between all other comorbidity comparisons, *p* < 0.05 was reported.

Linear regression was used to identify demographic and clinical factors that were significantly associated with NBM structural and functional imaging metrics in CN AC^−^. Factors that were significantly correlated with NBM structural or functional imaging metrics were controlled in further analyses.

One‐way analysis of covariance was used separately in CN and MCI matched samples to compare NBM GM densities and NBM FC between AC^−^ and AC^+^, controlled for identified demographic and clinical factors. The Benjamini–Hochberg procedure was applied to correct for the false discovery rate [30].

We assessed the association between TACB and cognition as well as the association of NBM metrics and cognition followed by mediation analysis using the PROCESS v3.1 macro (http://www.processmacro.org/index.html) for SPSS 21. The significance of indirect effects was tested using bootstrapping with 5000 replications. Mediation was accepted as having occurred if the indirect effect (x*y) was statistically significant.

Analysis of variance (ANOVA) tests were used to compare NBM GM density and FC between MCI and CN to confirm the expected sensitivity of the NBM imaging metrics to at‐risk or prodromal AD. To explore preferential AC effects, we computed effect sizes and 95% confidence intervals (CIs) in the various contrasts. We chose Cohen *f* to reflect the effect size, with *f* ≥ 0.4 considered a large, 0.25 ≤ *f* < 0.4 a medium, and *f* < 0.25 a small effect size [31].

We ran ANOVA tests to compare hippocampal GM, precuneus GM, visual FC network strength, and comorbidities between the respective AC medication strata.

To explore the reported differential susceptibility of *APOE* Ɛ4 carrier status to central AC effects [32, 33], as a further post hoc test, we used ANOVA to assess whether *APOE* Ɛ4 carriers status had a moderator effect on the effect of AC medication on NBM GM density, NBM FC, or cognition. in the whole study cohort, due to the small number of people with *APOE* Ɛ4 status.

## RESULTS

A total of 568 participants (mean age ± SD = 73.5 ± 7.8 years, age range = 55–97 years, 301 women [53.0%]) were included in this study (details of the whole cohort are given in Table S1). One hundred seventy‐eight (31%) participants were classed as AC^+^, with the majority taking one mild AC drug. The number of participants who had low, medium, or high TACB score was not significantly different between CN AC^+^ and MCI AC^+^ (*p* = 0.655).

The matched test sample included 319 participants (Table S1). CN AC^+^ compared to CN AC^−^ showed lower ADAS‐Cog score (*p* = 0.004), but did not differ in demographic factors, with a trend of fewer education years in CN AC^+^. No significant differences were seen for cognitive and demographic factors between MCI AC^−^ and MCI AC^+^.

### AC medication and NBM MRI metrics

To explore relevant covariates of no interest for NBM GM density, we assessed their association between demographic factors in all 246 CN AC^−^ participants with T1 data. No significant correlation was found between NBM GM density and age (*p* = 0.487). Nonetheless, based on prior reports on NBM atrophy over the age span [34], we chose to control for age in the analyses of NBM GM density.

**FIGURE 2:**
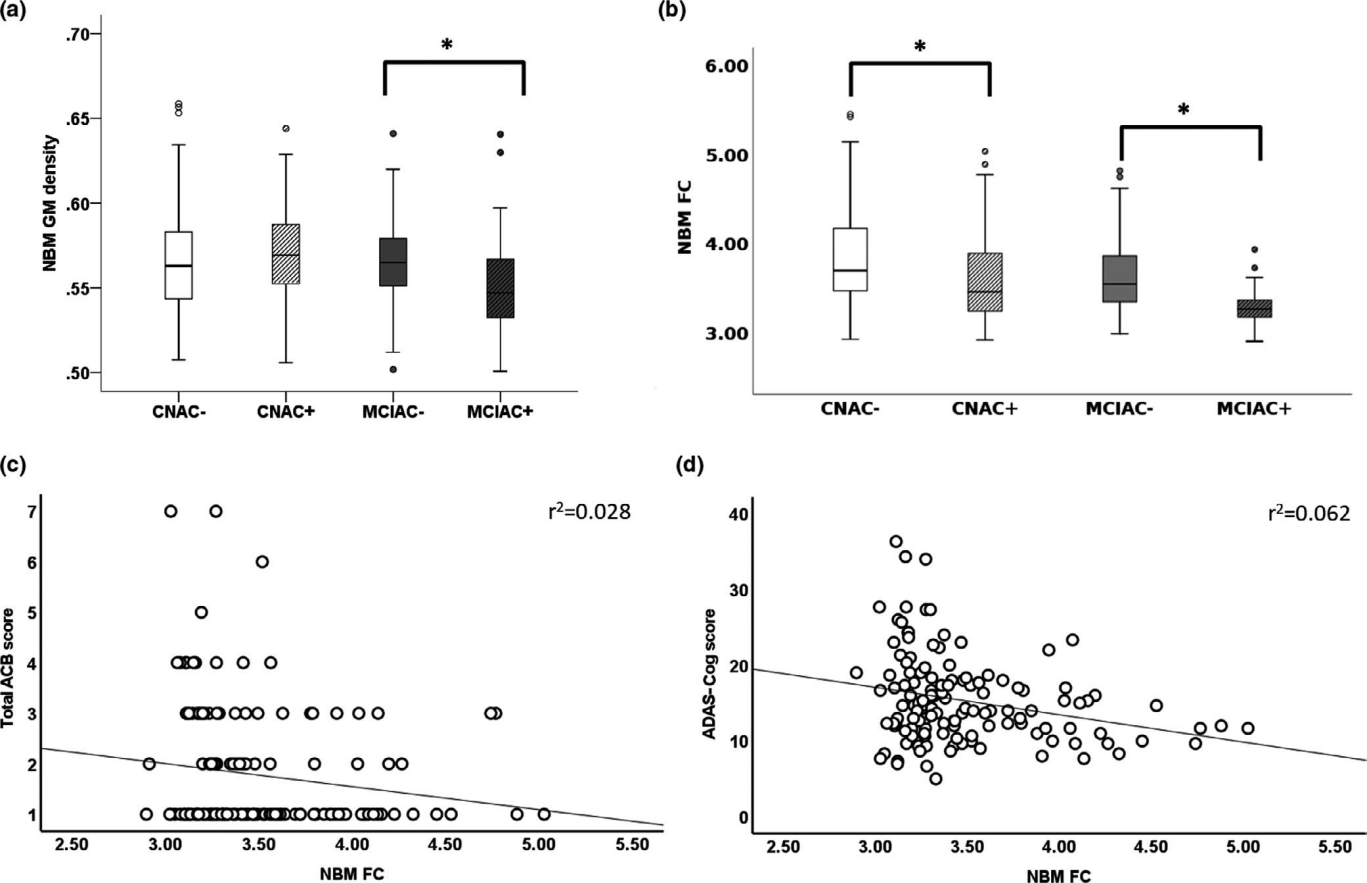
Anticholinergic drug use and nucleus basalis of Meynert (NBM) imaging metrics. Bar charts show the group differences of (a) NBM gray matter (GM) density and (b) NBM functional connectivity (FC) between cognitively normal (CN) anticholinergic (AC)^−^, CN AC^+^, mild cognitive impairment (MCI) AC^−^, and MCI AC participants. Boxplots display the value range of 25%–75% and the median values. (c) Scatterplots show the significant correlation between NBM FC and total AC burden (ACB) score. (d) Scatterplots show the significant correlation between NBM FC and Alzheimer's Disease Assessment Scale–Cognitive Subscale (ADAS‐Cog) score. Significance level was set at *p < 0.05

No significant difference of NBM GM density was found between CN AC+ and CN AC^−^ participants (*p* = 0.824). NBM GM density was reduced in MCI AC^+^ compared to MCI AC^−^, with a medium effect size (0.55 ± 0.03 vs. 0.56 ± 0.03, Cohen *f* = 0.26, 95% CI = 0.10–0.45, *p* = 0.002; corrected *p* < 0.05; Figure 2a). There was no correlation between NBM GM density and TACB in CN AC^+^ and MCI AC^+^.

**FIGURE 3:**
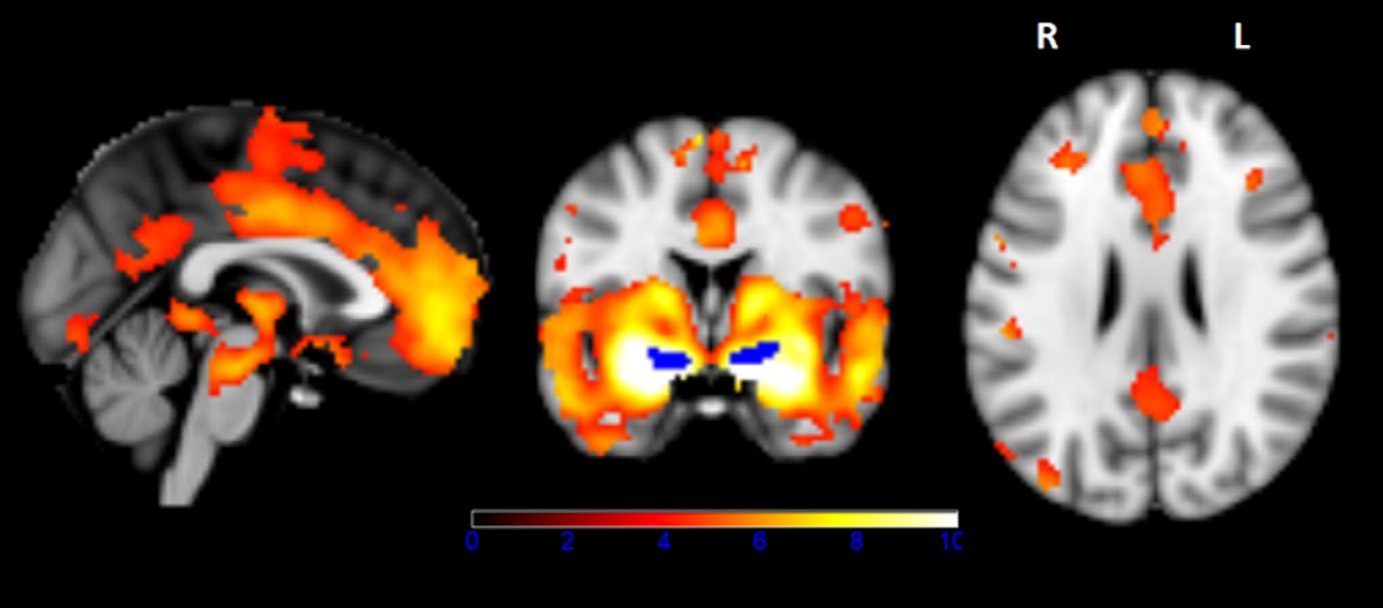
Nucleus basalis of Meynert (NBM) functional network reconstructed as seed‐based functional connectivity map of the NBM derived from the independent discovery subsample (*n* = 162). All results were masked by a gray matter template obtained from a Montreal Neurological Institute 152 standard space T1‐weighted average image (corrected *p* < 0.05). L, left; R, right

NBN functional network was reconstructed in the independent discovery sample. NBM was functionally connected with bilateral frontal cortex, anterior cingulate cortex, bilateral insula, bilateral thalamus, bilateral hippocampus, posterior cingulate cortex (PCC), and bilateral lateral occipital cortex (Figure 3, Table S2), which was similar to the NBM functional maps identified in our previous study [28] but with additional network areas in the PCC and bilateral thalami.

Education was significantly but weakly correlated with NBM FC *Z* score (*r* = 0.191, *p* = 0.001), and was hence controlled for in NBM FC tests, in addition to age, due to previous reports of age‐related changes of NBM FC [35].

There was a significant but small effect of AC medication use on NBM FC, with lower NBM FC observed in CN AC^+^ compared to CN AC^−^ (3.6 ± 0.5 vs. 3.9 ± 0.6, Cohen *f* = 0.24, 95% CI = 0.10–0.39, *p* = 0.001; corrected *p* < 0.05; Table 2, Figure 2b). MCI AC^+^ versus MCI AC^−^ also had lower NBM FC, with large effect size (3.29 ± 0.20 vs. 3.66 ± 0.46, Cohen *f* = 0.49, 95% CI = 0.30–0.68, *p* < 0.001; corrected *p* < 0.05; Table 2, Figure 2b). There was a trend correlation between NBM FC and TACB in CN AC^+^ (*r* = −0.200, *p* = 0.054) and MCI AC^+^ (*r* = −0.252, *p* = 0.059).

**TABLE 2:** Structural and functional measurements of imaging markers

Measurement	Cognitively normal				MCI				*p*
	Total, *n* = 193	AC^−^ participants, *n* = 97	AC^+^ participants, *n* = 96	*p*	Total, *n* = 126	AC^−^ participants, *n* = 67	AC^+^ participants, *n* = 59	*p*	
NBM GM density, mean voxel value (SD)^a^	0.57 (0.04)	0.57 (0.05)	0.57 (0.03)	0.824	0.56 (0.03)	0.56 (0.03)	0.55 (0.03)	0.002*	0.010*
NBM‐NBM network functional connectivity, mean (SD), *Z* score^b^	3.7 (0.5)	3.9 (0.6)	3.6 (0.5)	0.001*	3.48 (0.4)	3.66 (0.46)	3.29 (0.20)	<0.001*	<0.001*
Visual cortex functional connectivity, mean (SD), *Z* score^b^	5.1 (0.8)	4.9 (0.8)	5.1 (0.8)	0.254	4.9 (0.8)	4.8 (0.8)	5.0 (0.8)	0.388	0.188
Whole hippocampal GM volume, mean (SD), cm^3^	7.31 (0.88)	7.44 (0.80)	7.16 (0.95)	0.119	7.17 (0.90)	7.20 (0.86)	7.14 (0.96)	0.581	0.153
Whole precuneus GM volume, mean (SD), cm^3^	17.05 (2.43)	17.11 (2.45)	16.98 (2.41)	0.999	17.54 (2.16)	17.6 (2.2)	17.5 (2.1)	0.864	0.095

Abbreviations: AC, anticholinergic; ANOVA, analysis of variance; GM, gray matter; MCI, mild cognitive impairment; NBM, nucleus basalis of Meynert.

a Group comparison using ANOVA test controlled for age.

b Group comparison using ANOVA test controlled for age and education.

* Significant level at *p* < 0.05.

Secondary tests revealed that NBM GM density was significantly reduced in MCI compared with CN (0.57 ± 0.04 vs. 0.56 ± 0.03, Cohen *f* = 0.13, 95% CI = 0.03–0.25, controlled for age, *p* = 0.01). Lower NBM GM density in MCI compared to CN was seen in AC^+^ participants (Cohen *f* = 0.33, 95% CI = 0.17–0.50, *p* < 0.001; Table 2) but not in AC^−^ participants (*p* = 0.519). MCI participants displayed lower NBM FC compared to CN participants (3.48 ± 0.40 vs. 3.73 ± 0.54), controlled for education (Cohen *f* = 0.23, 95% CI = 0.12–0.35, *p* < 0.001). NBM FC was lower in MCI versus CN in both AC^−^ and AC^+^ strata, with small effect in AC^−^ (Cohen *f* = 0.17, 95% CI = 0.03–0.34, *p* = 0.016) and medium effect in AC^+^ (Cohen *f* = 0.36, 95% CI = 0.21–0.53, *p* < 0.001).

### NBM imaging metrics mediate the relationship between AC medication burden and cognition

All analyses were performed in the AC^+^ sample. TACB was weakly correlated with ADAS‐Cog (*r* = 0.168, *p* = 0.005; Figure 2c). No significant correlation was identified between NBM GM density and ADAS‐Cog (*p* = 0.645) or TACB (*p* = 0.450). ADAS‐Cog was weakly negatively correlated with NBM FC (*r* = −0.249, *p* = 0.005; Figure 2d). Hence, only NBM FC was tested as a potential mediator of the association between TACB and ADAS‐Cog. NBM FC modestly (indirect effect = 0.30, 95% CI = 0.16–0.49) mediated the association between TACB and ADAS‐Cog.

### Bias assessment of AC medication effects

No significant effect of AC medication was seen for CN or MCI strata on amyloid status or on hippocampal or precuneus GM volumes. There was no significant difference in hippocampal or precuneus volumes between AC medication strata for CN or MCI. No significant difference of primary visual cortex FC was found between CN AC^−^ and CN AC^+^ (*p* = 0.193) or between MCI AC^−^ and MCI AC^+^ (*p* = 0.388), making nonspecific and especially vascular confounds less likely.

The range of indications for AC medication prescriptions was reflected in higher prevalence of neurological, cardiovascular, respiratory, and gastrointestinal disorders in CN AC^+^ versus CN AC^−^, whereas only respiratory disorders were more common in MCI AC^+^. CN AC^+^ versus CN AC^−^ demonstrated a higher vascular risk profile (CMC score = 1.41 ± 1.16 vs. 1.13 ± 1.1, *p* < 0.001), but interestingly, no difference was seen between MCI AC^+^ and MCI AC^−^ participants, showing that the reported AC medication effects cannot be explained by differences in vascular risk profiles.

### Effects of *APOE* Ɛ4 carrier status

A further post hoc test was undertaken to explore differential susceptibility for AC effects between *APOE* genotypes in the whole study cohort. We did not find a significant moderator effect of *APOE* Ɛ4 carrier status on the effect of AC medication on forebrain MRI metrics or cognition NBM GM density (*p* = 0.100) with NBM FC (*p* = 0.654) or ADAS‐Cog score (*p* = 0.151).

## DISCUSSION

We provide the first evidence of structural and functional impairment of the NBM, a key cholinergic forebrain hub, linked to AC prescription in the ADNI Phase 3 (ADNI3) cohort, highlighting a possible mechanism of the reported elevated risk of dementia linked to AC medication. Importantly, the detrimental central cholinergic effects were demonstrated in both cognitive healthy and mild cognitively impaired participants with a prescription history of at least 1 year of drugs with mainly mild ACB. AC medication strata were matched for *APOE* Ɛ4 carrier or amyloid status and did not differ in established MRI AD markers, arguing against bias effect from preclinical AD and for a specific central cholinergic effect.

We found NBM GM density loss to be associated with the use of mild–moderate AC medication in MCI in a case–control design, carefully matched on demographics, *APOE*, and amyloid markers. This is the first NBM morphometric study on AC medication effects, but findings are generally in line with two previous MRI studies in CN on AC medication that reported increased brain atrophy and temporal cortical thinning [14, 36]. It has been suggested that global brain atrophy in AC medication may indirectly arise from affected central cholinergic pathways that may render the brain more vulnerable to stress‐related neurotoxicity. In our study, there was a non‐significant reduction of hippocampal GM volume, but no change was seen in precuneus GM volume, which would favour an increased cholinergic rather than general brain vulnerability. Selective vulnerability of the basal forebrain cholinergic neurons, and in particular those in Ch4 (NBM), to oxidative stress is well established [37]. Beyond oxidative stress, basal forebrain cholinergic neurons are also strongly dependent on target‐derived nerve growth factor (NGF) for preservation of cholinergic phenotype, which led to the notion of retrograde NBM atrophy in AD [38], but links between NGF and long‐term AC medication remain to be investigated.

We report reduced NBM GM density in MCI compared to CN, which is well in line with early cholinergic failure in MCI and previous studies demonstrating NBM atrophy [39]. Importantly, we observed NBM GM density loss in MCI with AC medication use compared to those without, providing the first evidence of accelerated NBM neuronal tissue injury associated with AC medication despite the absence of differences in the severity of cognitive impairment. These findings are further in support of a putative neurotrophic role of cholinergic tone orchestrated through the emerging understanding of modulated gene expression, translation, and cellular signalling cascades [40].

We also demonstrated detrimental effects of AC medication use on the NBM cholinergic network, in keeping with the hypothesized central hypocholinergic state. The NBM FC map was generated in an independent subsample of older participants using a Ch4/NBM template seed to increase anatomical specificity over expert manual seeds [28] which allowed robust reconstruction of the medial and lateral cholinergic pathways [41] similar to what was previously reported in a younger sample [35]. NBM FC was significantly reduced in CN and MCI AC^+^ compared to AC^−^. In parallel, we confirm previous reports that AC medication use is associated with impaired global cognitive performance in CN [12, 14, 25], but surprisingly not in MCI. We further showed that cholinergic network functional integrity partially mediated the association between AC medication use and global cognitive performance. These findings provide a potential biological basis for the impaired cognition associated with AC medication use through functional changes of the cholinergic network.

The effect size of AC medication on NBM imaging metrics was medium/large in MCI but absent/small in CN, suggesting a preferential vulnerability to AC in the at‐risk population, and the functional NBM network. This interpretation is further supported by the observed significant anticorrelation between NBM FC and ACB burden in MCI AC^+^, with only a trend association between NBM FC and ACB burden in CN AC^+^. Also, cognition was weakly correlated with NBM FC only in the MCI cohort. It is conceivable that MCI status and AC medication have superadditive effects on NBM impairment. In keeping with previous studies, we show significant cognitive cohort effects on the NBM imaging metrics with GM loss in MCI versus CN [42] and NBM FC reduction in MCI versus CN [21], and additionally report that the effect sizes of MCI status for both metrics were small in AC^−^ but large in AC^+^. Taken together, this suggests a more complex multifactorial interplay, which could lead to increased vulnerability of the central cholinergic pathways to AC medication in MCI with some preexisting NBM disruption.

Several limitations were noted in this study. First, according to the ADNI3 protocol, medication use was based on self‐report, which may lead to underreporting of AC medication use. There is also no accurate information of the duration of AC medication available in the ADNI3 study. Future studies using medical/prescription records would be needed to further characterize the specific medication effects. Second, we excluded participants using ChEIs, so we cannot comment on the degree to which AC‐related NBM effects may be reversible. Third, although the groups were well matched for demographics and AD risk and showed no differences in AD general MRI markers, due to the cross‐sectional nature, we cannot exclude biases from comorbidities and as expected with indications for commonly prescribed AC medication. We found a higher vascular risk factor score in CN AC^+^ versus CN AC^−^ and in general somewhat higher frequencies of cardiovascular, neurological, respiratory, and gastrointestinal comorbidities in AC^+^ subgroups. Last, despite the use of one of the largest well‐phenotyped imaging cohort studies in old age and dementia, we had limited power to study genotypic vulnerability, which may explain the lack of observed *APOE* Ɛ4 moderator effects.

## CONCLUSIONS

Our study provides new evidence of the detrimental effect of even mild–moderate AC medication use on NBM GM density in MCI and NBM functional network connectivity in CN and MCI. The link between NBM GM density loss and AC medication suggests accelerated NBM degeneration as a putative mechanism of the elevated risk of dementia. Impaired NBM FC in AC^+^ provides support for an AC medication‐induced central hypocholinergic state, which partially mediated the association between ACB and cognitive impairment. Moreover, a pattern of nominally stronger AC effects on NBM GM and FC changes in MCI points to a possible increased vulnerability of the central cholinergic pathways to AC medication in preexisting early cholinergic failure. Last, our findings highlight the suitability of the reported NBM imaging metrics as markers of central cholinergic failure for future mechanistic studies to inform on trials that may coprescribe ChEIs or deprescribe AC medication in the most at‐risk population to prevent dementia.

## CONFLICT OF INTEREST

The authors declare that they have no competing interests.

## AUTHOR CONTRIBUTIONS

**Dewen Meng:** Conceptualization (equal), data curation (equal), formal analysis (lead), investigation (equal), methodology (equal), project administration (supporting), resources (lead), software (lead), supervision (supporting), validation (equal), visualization (equal), writing–original draft (lead), writing–review & editing (equal). **Ali‐Reza Mohammadi‐Nejad:** Conceptualization (supporting), data curation (supporting), formal analysis (supporting), investigation (supporting), methodology (supporting), resources (supporting), software (supporting), validation (supporting), visualization (supporting), writing–original draft (supporting), writing–review & editing (equal). **Stamatios N. Sotiropoulos:** Conceptualization (supporting), data curation (supporting), formal analysis (supporting), investigation (supporting), methodology (supporting), resources (supporting), software (supporting), validation (supporting), visualization (supporting), writing–original draft (supporting), writing–review & editing (equal). **Dorothee P. Auer:** Conceptualization (equal), data curation (equal), formal analysis (equal), funding acquisition (lead), investigation (equal), methodology (equal), project administration (supporting), resources (supporting), software (supporting), supervision (lead), validation (equal), visualization (equal), writing–original draft (equal), writing–review & editing (lead).

## Data Availability

Data used in preparation of this article were obtained from the ADNI database (adni.loni.usc.edu). As such, the investigators within ADNI contributed to the design and implementation of ADNI and/or provided data but did not participate in analysis or writing of this report. A complete listing of ADNI investigators can be found at http://adni.loni.usc.edu/wp‐content/uploads/how_to_apply/ADNI_Acknowledgement_List.pdf.
