# Supplementary material for: Anticholinergic drugs and forebrain magnetic resonance imaging changes in cognitively normal people and those with mild cognitive impairment
Source: Eur J Neurol. 2022 Feb 7;29(5):1344–53. doi: 10.1111/ene.15251 (PMC9304308; doi:10.1111/ene.15251)
Supplement: Supplementary file 1 [file ENE-29-1344-s001.docx]

# Supplementary Material

**Appendix S1**

## Inclusion and exclusion criteria of cognitively normal and mild cognitive impairment participants

|  | Cognitively normal (CN) participants | Mild cognitive impairment (MCI) participants |
| --- | --- | --- |
| Inclusion criteria | - Participant with or without subjective memory complaints, verified by a study partner, beyond what one would expect for age - Normal memory function documented by scoring above education adjusted cutoffs on the Logical Memory II subscale (Delayed Paragraph Recall, Paragraph A only) from the Wechsler Memory Scale-Revised (the maximum score is 25):   a: ≥ 9 for 16 or more years of education  b: ≥ 5 for 8-15 years of education  c: ≥ 3 for 0-7 years of education   - Mini-Mental State Exam score between 24 and 30 inclusive (Exceptions may be made for participants with less than 8 years of education at the discretion of the Project Director) - Clinical Dementia Rating=0. Memory Box score must be 0 - Cognitively normal, based on an absence of significant impairment in cognitive functions or activities of daily living - Stability of Permitted Medications for at least 4 weeks:   a: Stable doses of antidepressants lacking significant anticholinergic side effects (if they are currently adequately treated for depressive symptoms and do not have a history of major depression within the past 1 years)  b: Estrogen replacement therapy is permissible  c: Gingko biloba is permissible, but discouraged  d: Washout from psychoactive medication (e.g., excluded antidepressants, neruoleptics, chronic anxiolytics or sedative hypnotics, etc.) for at least 4 weeks prior to screening | - Participant must express a subjective memory concern as reported by participant, or recalled by study partner or clinician - Abnormal memory function documented by scoring above education adjusted cutoffs on the Logical Memory II subscale (Delayed Paragraph Recall, Paragraph A only) from the Wechsler Memory Scale-Revised (the maximum score is 25):   a: < 11 for 16 or more years of education  b: ≤ 9 for 8-15 years of education  c: ≤ 6 for 0-7 years of education   - Mini-Mental State Exam score between 24 and 30 inclusive (Exceptions may be made for participants with less than 8 years of education at the discretion of the Project Director) - Clinical Dementia Rating=0.5. Memory Box score must be 0.5 - General cognition and functional performance sufficiently preserved such that a diagnosis of Alzheimer’s disease cannot be made by the site physician at the time of the Screening Visit - Stability of Permitted Medications for at least 4 weeks:   a: Stable doses of antidepressants lacking significant anticholinergic side effects (if they are currently adequately treated for depressive symptoms and do not have a history of major depression within the past 1 years)  b: Estrogen replacement therapy is permissible  c: Gingko biloba is permissible, but discouraged  d: Washout from psychoactive medication (e.g., excluded antidepressants, neruoleptics, chronic anxiolytics or sedative hypnotics, etc.) for at least 4 weeks prior to screening  e: cholinesterase inhibitors and memantine are allowable if stable for 12 weeks prior to Screening Visit |
| Inclusion criteria specific to newly enrolled participants   - Geriatric Depression Scale score less than 6 - Age between 55-90 years (inclusive) - Study partner who has frequent contact with the participant (i.e. minimum average of 10 hours per week) and is available to accompany the participant to all clinic visits for the duration of the protocol - Visual and auditory acuity adequate for neuropsychological testing - Good general health with no diseases expected to interfere with the study - Participant is not pregnant, lactating, or of childbearing potential (i.e. women must be two years post-menopausal or surgically sterile) - Willing and able to participate in a longitudinal imaging study - Modified Hachinski Ischaemic Score less than or equal to 4 - Completed six grades of education or has a good work history (sufficient to exclude mental retardation) - Must speak English or Spanish fluently - Willing to undergo repeated MRIs (3T) and at least two PET scans - Agrees to collection of blood for genomic analysis (including GWAS sequencing and other analysis), APOE testing and biospecimen banking - Agrees to collection of blood for biomarker testing - Agrees to at least one lumbar puncture for the collection of CSF - Agrees to share genomic data and biomarker samples   Inclusion criteria specific to rollover participants   - Must have been enrolled and followed in ADNI1, ADNIGO, or ADNI2 for at least one year - Willing and able to continue to participate in an ongoing longitudinal study. A reduced battery of tests is allowable if the participant is not able/willing to complete the full battery | | |
| Exclusion criteria | - Any significant neurologic disease, such as Parkinson’s disease, multi-infarct dementia, Huntington’s disease, normal pressure hydrocephalus, brain tumor, progressive supranuclear palsy, seizure disorder, subdural hematoma, multiple sclerosis, or history of significant head trauma followed by persistent neurologic deficits or known structural brain abnormalities | - Any significant neurologic disease other than suspected incipient AD, such as Parkinson’s disease, multi-infarct dementia, Huntington’s disease, normal pressure hydrocephalus, brain tumor, progressive supranuclear palsy, seizure disorder, subdural hematoma, multiple sclerosis, or history of significant head trauma followed by persistent neurologic deficits or known structural brain abnormalities |
| The following additional exclusion criteria apply to all diagnostic categories:   - Screening/Baseline MRI brain scan with evidence of infection, infarction, or other focal lesions or multiple lacunes or lacunes in a critical memory structure - Subjects that have any contraindications for MRI studies, including the presence of cardiac pacemakers, or metal fragments or foreign objects in the eyes, skin or body - Major depression, bipolar disorder as described in DSM-IV within the past 1 year. Psychotic features, agitation or behavioural problems within the last 3 months that could lead to difficulty complying with the protocol - Currently treated with medication for obsessive-compulsive disorder or attention deficit disorder - History of schizophrenia (DSM IV criteria) - History of alcohol or substance abuse or dependence within the past 2 years (DSM IV criteria) - Any significant systemic illness or unstable medical condition, which could lead to difficulty complying with the protocol - Clinically significant abnormalities in B12 or TFT2 that might interfere with the study. A low B12 is exclusionary, unless follow-up labs (homocysteine [HC] and methylmalonic acid [MMA]) indicate that it is not physiologically significant. - Residence in a skilled nursing facility - Current use of specific psychoactive medications (e.g., certain antidepressants, neuroleptics, chronic anxiolytics or sedative hypnotics). Current use of warfarin or other anticoagulants such as dabigatran, rivaroxaban and apixaban (exclusionary for lumbar puncture) - Current use of any other exclusionary medications - Investigational agents are prohibited one month prior to entry and for the duration of the trial - Participation in clinical studies involving neuropsychological measures being collected more than one time per year | | |

**
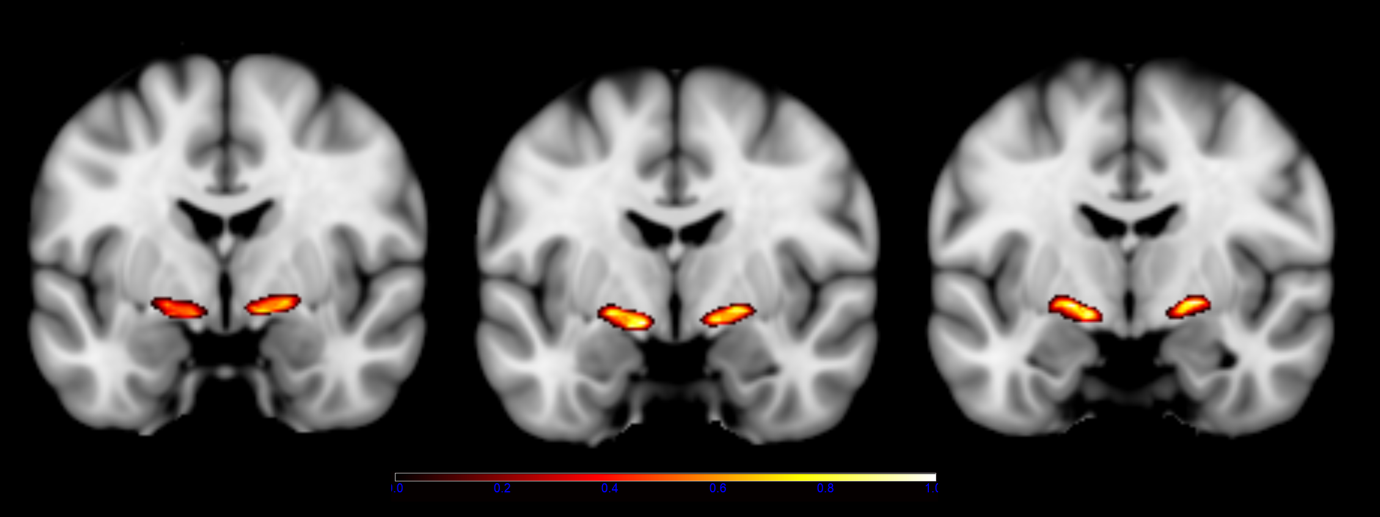
**

## Appendix S2 NBM (Ch4, red-yellow) probability map on three consecutive slices of T1-weighted images downloaded from the SPM 12 Anatomy Toolbox

**Appendix S3**

The probabilistic anatomical maps of NBM were thresholded at 28%[1] and registered to each participant’s T1-weighted images using a non-linear transformation. Participants’ GM tissue probability maps were extracted using FAST[2] in FMRIB Software Library (FSL) v6.0. To reduce partial volume effects and anatomically match Ch4, a threshold of 60% GM probability was applied. To choose the optimal GM threshold, we used the published Ch4 volume as a reference [1] and then used various GM thresholds (40%, 50% and 60%) to calculate the NBM GM volumes accordingly. We found that the results of NBM GM volume using the 60% GM threshold was the closest to the published Ch4 volume. To correct for brain size, we also extracted individual total intracranial volumes (TIV), which was automatically calculated using the CAT12 toolbox (http://www.neuro.uni-jena.de/cat/, version r914) within SPM12 (http://www.fil.ion.ucl.ac.uk/spm/software/spm12/) using MATLAB (R2017a) and was treated as a covariate of no interest in the analyses for investigating the association among NBM structural imaging metrics, AC medication use and cognition. Then each participant’s NBM mask was multiplied with their corresponding GM mask, resulting in a NBM mask of GM density in each participant’s T1 native space for subsequent intensity analysis. The mean NBM GM density of each participant was extracted using FSL tool.

NBM seed-based functional connectivity analysis was then undertaken in the matched test data using the same approach. All analyses were carried out using FMRI Expert Analysis Tool (FEAT) Version 6.00, part of FMRIB’s Software Library (FSL) Version 6.0.1 (FMRIB's Software Library, [www.fmrib.ox.ac.uk/fsl](http://www.fmrib.ox.ac.uk/fsl))[3, 4]. Each participant’s NBM GM mask in T1 native space was co-registered to fMRI space and then used as the NBM mask for FC analysis. Nuisance regressors were defined as signal averaged from masks of the central deep white matter (WM) (198cm^3^), signal averaged over the masks of ventricles (20cm^3^) and six parameters obtained by rigid body head motion correction, and then were used in the general linear model. The WM and ventricular masks were generated from each participant’s T1-weighted image using FMRIB's Automated Segmentation Tool (FAST)[2], part of FSL software and then co-registered into fMRI space. An in-house Matlab code was applied to generate the thresholded WM and ventricular masks. We did not use the mean global signal as a regressor to avoid introducing spurious anticorrelations[5]. Z statistical images of each individual were based on Z>2.3 and a corrected cluster significance threshold of P<0.05. Higher-level analysis was carried out using FLAME (FMRIB’s Local Analysis of Mixed Effects) [6] in FSL to generate a template of NBM functional network map. All results were masked by grey matter masks obtained from the MNI152 template. To generate the template of NBM functional network map by using FLAME, Z statistical images of voxel-based high-level analyses were estimated based on Z greater than 2.3 and a familywise error-corrected cluster significance thresholded of P<0.05. The group-level GM masked NBM FC map was then binarised to yield the NBM cholinergic network template.

1. Zaborszky, L., et al., *Stereotaxic probabilistic maps of the magnocellular cell groups in human basal forebrain.* Neuroimage, 2008. **42**(3): p. 1127-41.

2. Zhang, Y.Y., M. Brady, and S. Smith, *Segmentation of brain MR images through a hidden Markov random field model and the expectation-maximization algorithm.* Ieee Transactions on Medical Imaging, 2001. **20**(1): p. 45-57.

3. Woolrich, M.W., et al., *Temporal autocorrelation in univariate linear modeling of FMRI data.* Neuroimage, 2001. **14**(6): p. 1370-86.

4. Woolrich, M.W., et al., *Multilevel linear modelling for FMRI group analysis using Bayesian inference.* Neuroimage, 2004. **21**(4): p. 1732-1747.

5. Cottam, W.J. and D.P. Auer. *Group differences in default mode network connectivity not just anti-correlation depend on choice of nuisance regressor model*. in *The International Society for Magnetic Resonance in Medicine (ISMRM)*. 2017.

6. Jezzard, P., P.M. Matthews, and S.M. Smith, *Functional MRI : an introduction to methods*. 2001, Oxford: Oxford University Press. xiii, 390 p., 22 p. of col. plates.

## Table S1 Demographic, clinical and cognitive information in the whole cohort

| **Characteristic** | **Cognitively normal (n=344)** | | | | **MCI (n=224)** | | | | **P^c^** |
| --- | --- | --- | --- | --- | --- | --- | --- | --- | --- |
|  | **Total (n=344)** | **AC- participants (n=246)** | **AC+ participants (n=98)** | **P^a^** | **Total (n=224)** | **AC- participants (n=144)** | **AC+ participants (n=80)** | **P^b^** |  |
| **Age, mean (SD), years** | 73.3 (7.8) | 72.6 (7.6) | 75.1 (7.9) | 0.010* | 73.8 (7.9) | 73.4 (7.8) | 74.6 (8.2) | 0.275 | 0.445 |
| **Female, n (%)** | 199 (57.8) | 138 (56.1) | 61 (62.2) | 0.334 | 102 (45.5%) | 60 (41.7%) | 42 (52.5%) | 0.126 | 0.005* |
| **Education, mean (SD), years** | 16.8 (2.3) | 16.9 (2.2) | 16.3 (2.4) | 0.017* | 16.7 (2.5) | 16.8 (2.5) | 16.5 (2.4) | 0.349 | 0.727 |
| **ADAS-cog, mean (SD)** | 13.1 (4.7) | 12.5 (4.4) | 14.4 (5.3) | 0.006* | 16.5 (6.0) | 16.5 (5.7) | 16.7 (6.5) | 0.832 | <0.001* |
| **APOE Ɛ4 carriers, n (%)** | 82 (32.4) | 67 (34.9) | 15 (24.6) | 0.158 | 47 (34.3%) | 31 (36.9%) | 16 (30.2%) | 0.464 | 0.736 |
| **Amyloid positive, n (%)** | 65 (35.5) | 42 (33.9) | 23 (39.0) | 0.513 | 44 (40.0%) | 25 (36.8%) | 19 (45.2%) | 0.426 | 0.456 |
| **Vascular risk, CMC score, mean(SD)** | 1.08 (1.0) | 0.95 (0.9) | 1.41 (1.2) | 0.001* | 1.2 (0.9) | 1.2 (0.9) | 1.4 (0.9) | 0.143 | 0.075 |
| **Total ACB scale, median (range)** | - | - | 1 (1-7) | - | - | - | 1 (1-7) | - | 0.483 |
|  | | | | | | | | | |
| **Psychiatric** | 84 (24.6) | 50 (20.6) | 34 (34.7) | 0.008* | 88 (39.8%) | 51 (35.7%) | 37 (47.4%) | 0.059 | <0.001* |
| **Neurologic (other than cognitive disorder)** | 111 (32.6) | 63 (25.9) | 48 (49.0) | <0.001* | 107 (48.4%) | 61 (42.7%) | 46 (59.0%) | 0.015* | <0.001* |
| **Head, Eyes, Ears, Nose, Throat** | 213 (62.5) | 142 (58.4) | 71 (72.4) | 0.019* | 138 (62.4%) | 85 (59.4%) | 53 (67.9%) | 0.135 | 0.533 |
| **Cardiovascular** | 227 (66.6) | 148 (60.9) | 79 (80.6) | 0.001* | 155 (70.1%) | 94 (65.7%) | 61 (78.2%) | 0.036* | 0.405 |
| **Respiratory** | 67 (19.6) | 37 (15.2) | 30 (30.6) | 0.002* | 71 (32.1%) | 39 (27.3%) | 32 (41.0%) | 0.027* | 0.001* |
| **Hepatic** | 12 (3.5) | 9 (3.7) | 3 (3.1) | 0.531 | 8 (3.6%) | 4 (2.8%) | 4 (5.1%) | 0.297 | 0.561 |
| **Dermatologic-Connective Tissue** | 119 (34.9) | 83 (34.2) | 36 (36.7) | 0.707 | 73 (33.0%) | 46 (32.2%) | 27 (34.6%) | 0.411 | 0.716 |
| **Musculoskeletal** | 248 (72.7) | 168 (69.1) | 80 (81.6) | 0.022* | 157 (71.0%) | 100 (69.9%) | 57 (73.1%) | 0.370 | 0.701 |
| **Endocrine-Metabolic** | 174 (51.0) | 119 (49.0) | 55 (56.1) | 0.281 | 114 (51.6%) | 69 (48.3%) | 45 (57.7%) | 0.115 | 0.931 |
| **Gastrointestinal** | 176 (51.6) | 113 (46.5) | 63 (64.3) | 0.004* | 116 (52.5%) | 64 (44.8%) | 52 (66.7%) | 0.001* | 0.863 |
| **Hematopoietic-Lymphatic** | 38 (11.1) | 29 (11.9) | 9 (9.2) | 0.570 | 28 (12.7%) | 21 (14.7%) | 7 (9.0%) | 0.291 | 0.594 |
| **Renal-Genitourinary** | 156 (45.7) | 104 (42.8) | 52 (53.1) | 0.093 | 98 (44.3%) | 66 (46.2%) | 32 (41.0%) | 0.277 | 0.795 |
| **Allergies or Drug Sensitivities** | 122 (35.8) | 78 (32.1) | 44 (44.9) | 0.034* | 75 (33.9%) | 42 (29.4%) | 33 (42.3%) | 0.037* | 0.717 |
| **Smoking, Alcohol Use, and/or Drug Use** | 27 (7.9) | 15 (6.2) | 12 (12.2) | 0.076 | 32 (14.5%) | 16 (11.2%) | 16 (20.5%) | 0.048* | 0.016* |
| **Malignancy** | 74 (21.7) | 48 (19.8) | 26 (26.5) | 0.192 | 30 (13.6%) | 21 (14.7%) | 9 (11.5%) | 0.332 | 0.019* |

a:Group comparison using T test between AC- CNs and AC+ CNs

b:Group comparison using T test between AC- MCI participants and AC+ MCI participants

c:Group comparison using T test between CNs and MCI participants

d:290 CNs and 161 MCI participants had ADAS-cog score information

e:253 CNs and 137 MCI participants had genotype information

f:183 CNs and 110 MCI participants had amyloid PET information

*: Significant level at P<0.05

## Table S2 Brain regions where NBM are functionally connected in the brain

|  | Coordinates (mm) | | | Z value |
| --- | --- | --- | --- | --- |
|  | *X* | *Y* | *Z* |  |
| Left superior frontal gyrus | -28 | 42 | 21 | 5.73 |
| Right superior frontal gyrus | 9 | 54 | 32 | 3.90 |
| Posterior cingulate cortex | 7 | -58 | 17 | 5.76 |
| Anterior cingulate cortex | 2 | 52 | -6 | 7.33 |
| Left thalamus | -7 | -13 | 2 | 6.65 |
| Right thalamus | 7 | -11 | 0 | 7.42 |
| Left insula | -48 | 20 | -12 | 7.50 |
| Right insula | 45 | 22 | -12 | 6.94 |
| Left hippocampus | -22 | -26 | -12 | 8.01 |
| Right hippocampus | 29 | -26 | -20 | 8.25 |
| Left medial temporal lobe | -53 | 1 | -25 | 6.71 |
| Right medial temporal lobe | 54 | -5 | -16 | 7.51 |
| Left lateral occipital cortex | -58 | -22 | -9 | 7.04 |
| Right lateral occipital cortex | 60 | -29 | -5 | 6.73 |
| Left nucleus basalis of Meynert | -21 | -1 | -19 | 11.97 |
| Right nucleus basalis of Meynert | 17 | -1 | -17 | 10.77 |
